# Deep Brain Stimulation and Microelectrode Recording for the Treatment of Parkinson’s Disease

**DOI:** 10.7759/cureus.27887

**Published:** 2022-08-11

**Authors:** Joshua Fejeran, Frank Salazar, Cesia M Alvarez, Faisal R Jahangiri

**Affiliations:** 1 Neuroscience, School of Behavioral and Brain Sciences, University of Texas at Dallas, Richardson, USA; 2 Neurophysiology, Global Innervation LLC, Dallas, USA; 3 Intraoperative Neurophysiological Monitoring Program, Labouré College of Healthcare, Milton, USA; 4 Neurology, AINeuroCare Academy, Dallas, USA

**Keywords:** neural impulse generator, dopaminergic neurons, pedunculopontine nucleus, striatum, globus pallidus interna, subthalamic nucleus, microelectrode recording, microelectrode placement, deep brain stimulation, parkinson’s disease

## Abstract

Parkinson’s disease (PD) is a neurological disorder in which nigrostriatal pathways involving the basal ganglia experience a decrease in neural activity regarding dopaminergic neurons. PD symptoms, such as muscle stiffness and involuntary tremors, have an adverse impact on the daily lives of those affected. Current medical treatments seek to decrease the severity of these symptoms. Deep brain stimulation (DBS) has become the preferred safe, and reliable treatment approach. DBS involves implanting microelectrodes into subcortical areas that produce electrical impulses directly to high populations of dopaminergic neurons. The most common targets are the subthalamic nucleus (STN), and the basal ganglia's globus pallidus pars interna (GPi). Research studies suggest that DBS of the STN may cause a significant reduction in the daily dose of L-DOPA compared to DBS of the GPi. DBS of the STN has suggested that there may be sweet spots within the STN that provide hyper-direct cortical connectivity pathways to the primary motor cortex (M1), supplementary motor area (SMA), and prefrontal cortex (PFC). In addition, the pedunculopontine nucleus (PPN) may be a new target for DBS that helps treat locomotion problems associated with gait and posture. Both microelectrode recording (MER) and magnetic resonance imaging (MRI) are used to ensure electrode placement accuracy. Using MER, stimulation of the STN at high frequencies (140<) decreased oscillatory neuronal firing by 67%. This paper investigates methods of intraoperative neuromonitoring during DBS as a form of PD treatment.

## Introduction and background

Description of the pathology related to Parkinson’s disease

Parkinson’s disease (PD) is a neurodegenerative disease that is characterized by uncontrollable and involuntary body movements (tremors and dyskinesias) [1]. Some other PD symptoms include a lack of balance, rigidity in the upper or lower limbs and trunk, and slow movement (bradykinesia). Additionally, the organization Parkinson’s Foundation explains that more than 60,000 Americans are diagnosed annually with PD and around 10 million individuals worldwide. Furthermore, it emphasizes the prevalence of men for PD and that older populations are at a higher risk of suffering from this debilitating neurological disease than younger ones [2]. Moreover, the genetic background and environmental factors involving each patient are factors heavily involved in the onset and development of this neurological disease [3].

The pathological nucleus of PD resides in the pars compacta of the substantia nigra. This area of the ventral midbrain is fully involved in the production and management of dopamine - a crucial neurotransmitter that is heavily involved in voluntary, fine body movements through the communication with the basal ganglia via the nigrostriatal dopaminergic pathway [1,3]. When populations of dopaminergic neurons start to decline in the pars compacta of the substantia nigra, dopamine levels start to lower (reuptake of DA as well), thus becoming the initial and critical step in the development of the typical symptoms of PD [1,3].

Even though this neurodegenerative disease has been heavily investigated throughout the years, and there have been major developments in medical treatments and different therapies, PD has proven to be a difficult and resilient disease to treat. The most commonly prescribed treatment for PD is dopamine supplementation through Levodopa [1]. Levodopa (L-DOPA) (L-3,4-dihydroxyphenylalanine) is a DA precursor that is typically administered orally [1,3]. Although levodopa has shown significant positive outcomes in patients suffering from PD, it does not represent a long-term solution for the total control or eradication of these terrible symptoms [1]. According to different studies, new investigations and treatments surrounding PD focus primarily on avoiding or slowing down the deterioration of dopaminergic neurons and pathways [1]. Moreover, other treatments, some of which have been primarily tested in animal models, such as NMDA and AMPA receptor antagonists, appear to decrease stiffness [3]. Also, some surgical procedures such as pallidotomy, insertions of DA neurons in the intrastriatal pathways, and mesencephalic fetal grafts containing DA neurons are examples of invasive treatments that have shown some success in the treatment of PD symptoms [3].

Deep brain stimulation (DBS) is a treatment for PD. This modern medical technique was introduced in 1997, becoming an alternative to other commonly used treatments for PD, such as thalamotomy and pallidotomy [4,5]. Thalamotomy is a surgical medical treatment in which a lesion is made on a specific and very small part of the thalamus [6]. Pallidotomy is another surgical treatment for PD that involves a lesion but also the globus pallidus [6]. Although the main goal of these treatments was to decrease the intensity of some of the symptoms of PD, such as tremors and stiffness, thalamotomy and pallidotomy come with several complications and side effects that have made these treatments less popular throughout the years [7]. Even though DBS treatment is still under constant medical investigation, it is a positive alternative that is more likely to produce significant rates of efficacy for the improvements in treating some of the most common PD symptoms compared to thalamotomy and pallidotomy, and DBS comes with a significantly fewer risks and side effects [4,7].

The therapeutic surgical process by which DBS performs is through electrodes placed in target subcortical areas such as the subthalamic nucleus (STN) that elicit constant electrical stimulation under heavily controlled settings (e.g., pulse width, amplitude, etc.) that are connected to a neurostimulator [4]. As a continuum of high-frequency pulses (approx. 100-180 Hz) are elicited through the targeted subcortical brain area, an inhibitory effect is generated, which is the main factor responsible for decreasing some PD symptoms [4,8]. Its reversibility and adjustability to every patient’s clinical situation are some of the benefits of DBS compared to other more invasive PD treatments such as thalamotomy. Furthermore, throughout extensive scientific investigations, it has been found that DBS can also be used to treat other types of illnesses depending on which subcortical area will be stimulated. According to the literature, DBS has offered significant results in treating epilepsy (e.g., one of the main target areas - anterior nuclei of the thalamus, etc.), movement disorders (target area - basal ganglia), and obesity (e.g., one of the main target areas - anterior hypothalamus), etc. [4].

Although DBS has become a widely available and commonly used medical treatment for PD, crucial external factors play important roles for the outcome of the stimulation to be successful. Patient characteristics such as age, the grade of the advancement of the disease, and cognitive impairment are considered [5]. Additionally, experienced surgeons, the usage of intra- and post-operative imaging techniques such as computed tomography (CT) and magnetic resonance imaging (MRI), and the need for more than one surgery are important factors to take into consideration for DBS to produce substantial positive results [5].

## Review

This review article aims to investigate the roles different types of intraoperative neuromonitoring play in the implantation, assessment, functioning, and successful utilization of DBS to treat PD. Also, we sought to explore more in-depth the origin and development of this neurodegenerative disease and to investigate other therapeutic routes that are still being used for the treatment of PD. 

PD, installation of DBS device and targeted spots for stimulation

**Figure 1:**
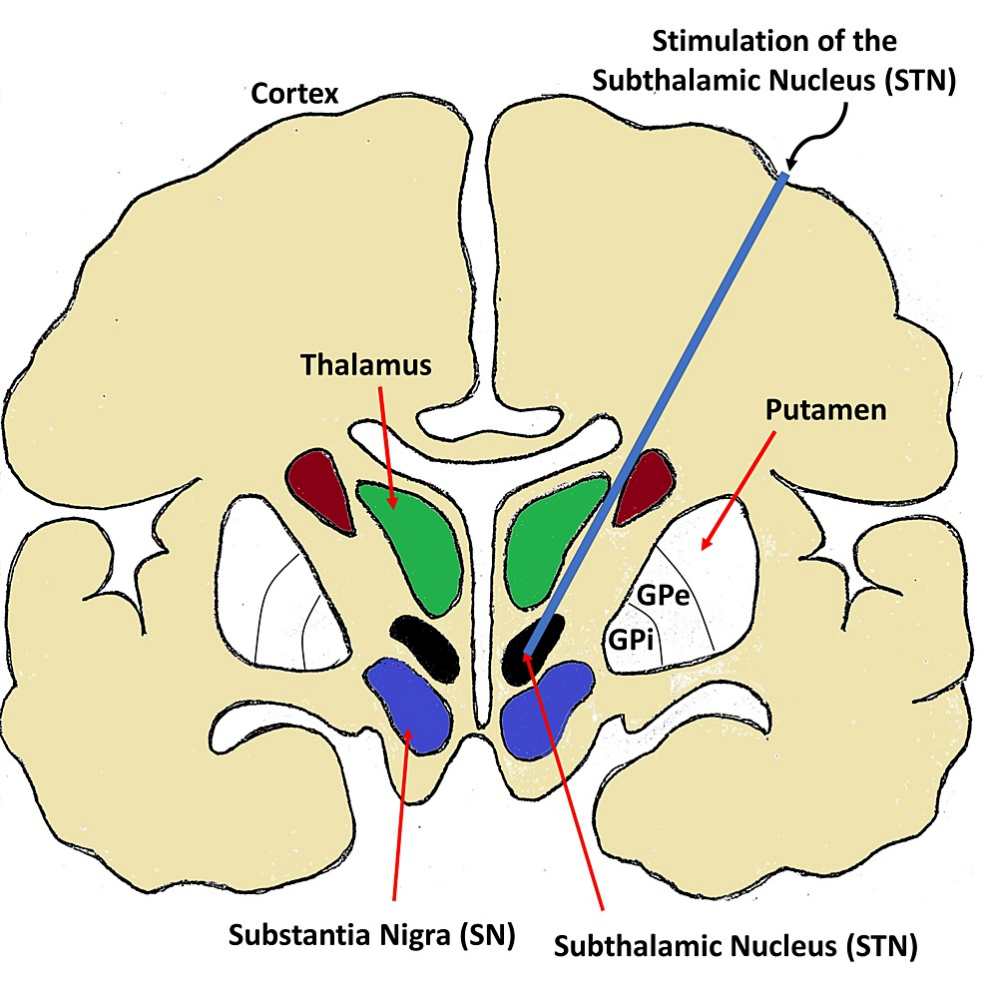
Electrode implantation for deep brain stimulation for targeting subthalamic nucleus (STN).

**Figure 2:**
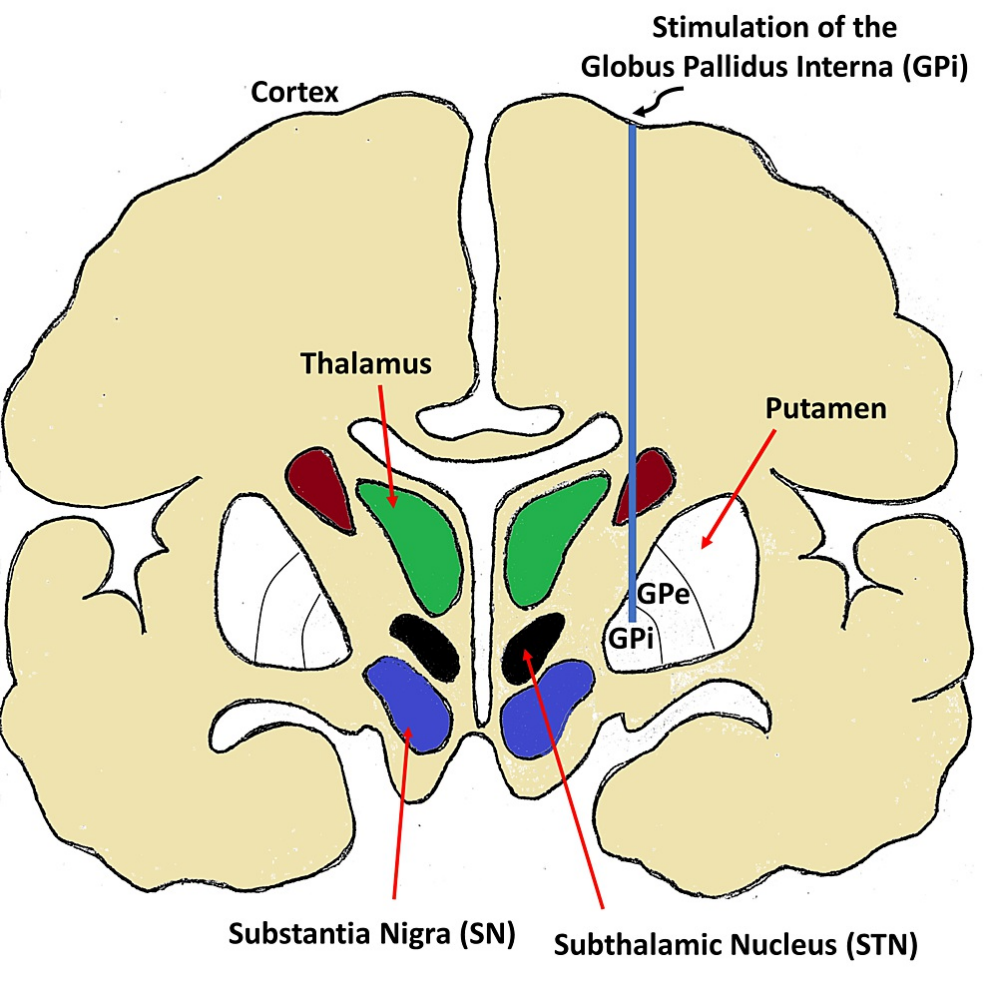
Electrode implantation for deep brain stimulation for targeting the globus pallidus interna (GPi).

**Figure 3:**
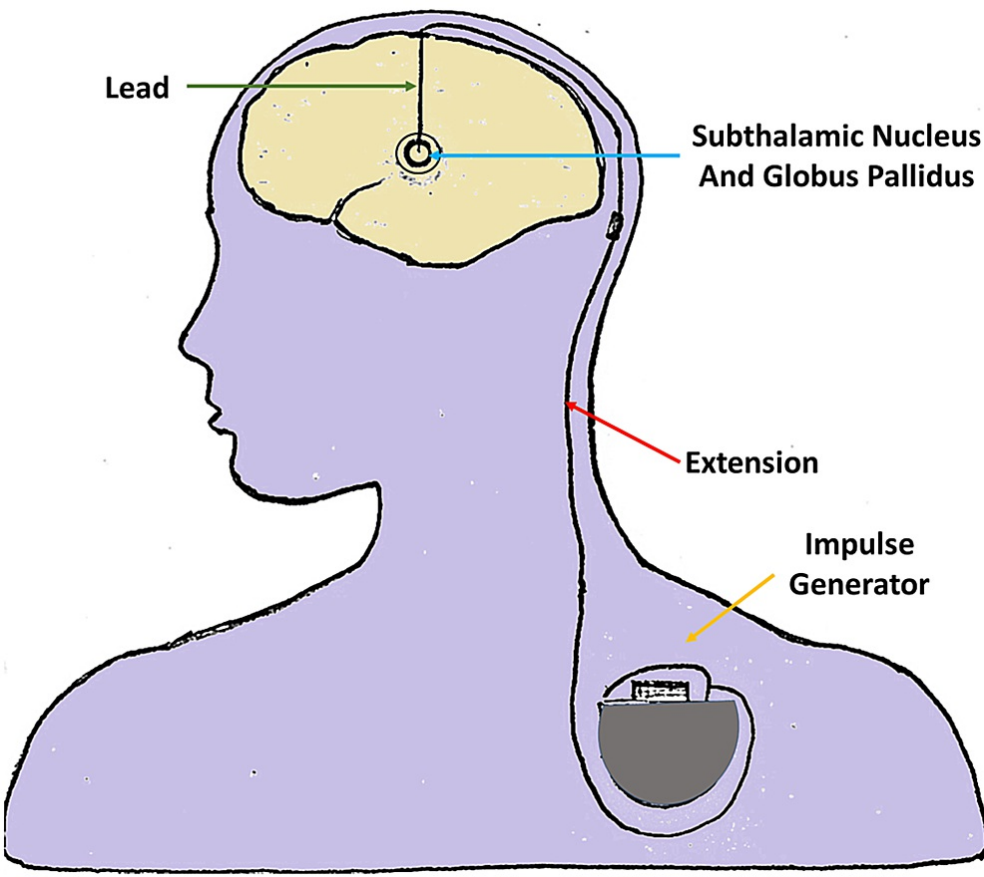
Impulse generator/neurotransmitter implanted and connected to the lead via an extension for deep brain stimulation (DBS).

Patients treated with DBS for PD must first undergo a two-part procedure to install the necessary devices for the treatment. First, one procedure is performed to insert electrodes onto a targeted brain area for stimulation. Traditionally, the electrode has been inserted into one of two possible locations: the STN (Figure 1) or the globus pallidus interna (GPi) (Figure 2). Both the STN and GPi are nuclei within the basal ganglia deep within the brain. The electrode is connected to an insulated wire inserted underneath the skin down to a position beneath the neck to the collarbone area. Afterward, a second procedure is performed, usually a few weeks later, and an impulse generator battery is installed under the collarbone. The impulse generator is then connected to the insulated wire and lead electrode, providing the electrical impulse to a part of the brain involved in motor function (Figure 3). Patients who undergo DBS are given a remote controller to turn the impulse generator device on or off. The installation of the electrodes within the brain and the impulse generator must be performed in two separate procedures. This cannot be accomplished in one procedure because physicians must use MRI and recordings of the brain cell activity to determine if the electrode is placed correctly on the targeted area of the brain (STN or GPi).

The goal of DBS is to disrupt the problematic electrical signals coming from those targeted areas of the brain. The specific outcome sought using DBS is treating symptoms such as tremors, rigidity, stiffness, slowed movement, and slowed walking. Notably, DBS is only a form of treatment to minimize or eliminate the movement disorders related to PD. However, DBS does not treat the other symptoms of PD that are associated with non-motor deficiencies such as depression, anxiety, apathy, mood disorders, pain, fatigue, speech deficits, swallowing issues, cognitive problems, memory problems, digestive problems, dementia, imbalance, sleep disorders, or freezing of gait. The electrical stimulation is delivered to areas in the brain that control movement by the impulse generator (Figure 3) and blocks abnormal nerve signals that cause tremors, rigidity, and bradykinesia. DBS is not a cure for PD, and it will not stop the progression of PD. DBS is found to be very helpful for some patients and not helpful at all for other patients. It is merely a treatment option that is considered to help alleviate some of the motor symptoms. Patients who respond well to L-dopa are candidates for DBS once the on-off phenomenon has started, or dyskinesias appear as a side effect of long-term L-dopa therapy.

According to clinical research studies, DBS changes individual neuron firing rates and patterns in the basal ganglia. The electrical current acts at synapses and triggers neighboring astrocytes to release a wave of calcium and promote the local release of neurotransmitters adenosine and glutamate. DBS therapy has also been proposed to increase blood flow and stimulate neurogenesis [9]. The local effects of deep-brain stimulation seem to show inhibition of neuronal cell bodies (-) and excitation of neighboring axons (+) where astrocytes release calcium which possibly leads to a release of glutamate (GLUT) and adenosine (ADO) while simultaneously increasing cerebral blood flow [9].

A burr hole is drilled in the skull to insert microelectrodes and a quadripolar deep-brain stimulation electrode [9]. Microelectrodes can be used to obtain electrophysiological recordings, which may reveal characteristic neuronal signals of the specific brain target. DBS has broad effects on neural networks due to its local influences, and DBS modulates the feedback loops that ultimately determine motor control. It is unclear whether there are definite advantages or disadvantages in selecting one target over another for DBS when considering electrode placement on the STN or GPi. According to four randomized, controlled clinical trials of DBS comparing results from microelectrode placement on the STN or GPi, there were no significant differences in improvement in motor function between the two study groups. However, in the larger trials, long-term follow-up showed a more rapid decline in cognitive function with treatment targeting the STN than the treatment targeting the GPi [9].

The putamen, caudate nucleus, and ventral striatum have somewhat different functions because of their participation in different basal ganglia loops [10]. The putamen receives most of its inputs from the motor and somatosensory areas of the cortex and projects by way of the globus pallidus and thalamus to the motor, premotor, and supplementary motor areas (SMAs). Therefore, the putamen is most likely involved in most of the motor functions of the basal ganglia. The caudate nucleus receives most of its inputs from association areas of the cortex and projects by way of the globus pallidus interna, substantia nigra pars reticularis, and the thalamus mostly to prefrontal areas [10].

Therefore, the caudate nucleus is likely more involved in cognitive functions and less directly in movement. The two segments of the globus pallidus have similar inputs but distinctly different sets of outputs. The external segment (GPe) receives inhibitory inputs from the striatum, and excitatory inputs from the STN, where it distributes widespread inhibitory (GABA) outputs to most other parts of the basal ganglia [10]. Furthermore, the internal segment (GPi) and the substantia nigra pars reticularis (SNr) receive inhibitory inputs from the striatum and excitatory inputs from the STN. The GPi projects mainly to the thalamus with the lenticular fasciculus going directly through the internal capsule and passing medially as a sheet of fibers between the STN and zona incerta, where it makes a turn in a posterolateral direction and enters the thalamus; however, the second collection of fibers from the GPi loops around the medial edge of the internal capsule as the ansa lenticularis where it joins the lenticular fasciculus and GABAergic fibers from the SNr in the thalamic fasciculus which then enters the thalamus [10].

Most of the efferent fibers from GPi-SNr to the thalamus also have a branch that descends to an area of the midbrain reticular formation adjacent to the decussation of the superior cerebellar peduncles known as the pedunculopontine nucleus (PPN) [10]. The STN is located across the internal capsule from the globus pallidus [10]. The STN provides a powerful excitatory input to the GPi-SNr neurons and is thought to have important effects on the pattern of inhibitory output from the basal ganglia. Major inputs to the STN from the cerebral cortex provide the most rapid access to basal ganglia output nuclei (cortex → STN → GPi), and different sectors of the STN deal with motor, cognitive, and affective functions. The substantia nigra is divided into two parts: pars compacta (SNc) and pars reticularis (SNr). The SNc has densely packed dopaminergic neurons blanket the basal ganglia with modulatory inputs. The SNr has loosely packed dopaminergic neurons and is the major output for the basal ganglia. The SNc and ventral tegmental area (VTA) project systemically to all areas of the striatum by fine axons that use dopamine as their neurotransmitter. Destruction of this nigrostriatal pathway is the underlying cause of PD [10]. The objective of DBS is to stimulate the areas within the basal ganglia that are firing irregularly to cause motor dysfunction and interrupt this irregular signaling to promote relief of motor symptoms associated with PD. Various studies have been conducted to evaluate the comparative results of DBS through stimulating the STN vs. GPi.

According to Akram et al., stimulation of the STN could elicit stimulation clusters or sweet spots that predict maximum improvement in rigidity, bradykinesia, and tremors through hyper-direct cortical connectivity in PD [11]. This study acquired high angular resolution diffusion imaging in 20 patients with advanced PD before bilateral STN DBS. Clusters corresponding to maximum improvement in tremor were in the posterior, superior, and lateral portion of the STN, whereas clusters corresponding to improvement in bradykinesia and rigidity were near the superior border in a further medial and posterior location. Using diffusion-weighted imaging (DWI) and probabilistic tractography for the left and right hemispheres, there were three cortical target areas were analyzed: (1) primary motor cortex (M1), (2) SMA, and (3) prefrontal cortex (PFC). Researchers generated a connectivity matrix between all the seed points in the combined total VTA area mask and all points in the cortical target masks (M1, SMA, and PFC) using the output from tractography [11].

Voxel-based statistical analysis of volumes of tissue activated was analyzed at increasing amplitudes one year after the DBS in the STN used in these 20 patients with advanced PD to map out statistically significant clusters in the STN area reflecting efficacy and side effects zones to generate probabilistic tractography streamlines (hyper-direct pathways) from these observed volumes to predefined cortical areas (M1, SMA, and PFC), and to identify the pattern of cortical connectivity that predicts response to DBS treatment of the STN [11].

Researchers acquired 270 diffusion scans per patient (in 2 x 128 direction sets) over 62 minutes, and the effect of STN-cortical connectivity was examined. The researchers hypothesized that the DBS of the STN exerted an effect through the hyper-direct pathway, and their results suggest that three hyper-direct pathways connect the combined electrode stimulation area in and around the STN. According to their research analysis, (1) connectivity to M1 appears to predict improvement in tremors, (2) connectivity to SMA appears to predict improvement in bradykinesia, and (3) connectivity to both SMA and PFC appears to predict improvement in rigidity. The conclusion from this research panel suggests the optimal DBS site for PD patients with tremors, bradykinesia, and rigidity appears to correspond to different areas in the motor STN. Stimulation in the central portion of the superior STN is most effective for tremors, whereas stimulation in further medial and posterior areas within the superior portion gives the highest improvements in bradykinesia and rigidity [11].

In a multicenter study of 69 patients with advanced PD, bilateral DBS of the STN (n = 49) or GPi (n = 20) was recorded and analyzed to determine the various effects of stimulating either STN or GPi with respect to “off” and “on” times with dyskinesia [12]. The multicenter study aimed to assess the efficacy of bilateral DBS in a large group of patients followed for a time frame between three to five years. The surgical target (STN or GPi) was not randomized but decided by each medical team according to their best clinical judgment at the time of recruitment. Patients were assessed pre-operatively for the baselines one year after DBS surgery and then again 3-4 years after DBS surgery. Evaluations were performed by neurologists specialized in movement disorders, and assessments were conducted pre-operatively in the poor mobility (“off” medication) and good mobility (“on” medication) states [12].

Bilateral STN stimulation induced a significant improvement of 50% (P = 0.00001) in the “off” medication UPDRS-III score with respect to baseline [12]. Bilateral GPi stimulation induced a significant improvement of 39% (P < 0.0001) in the “off” medication UPDRS-III score with respect to baseline. The motor scores in the “off” state were significantly reduced by stimulation, dramatically reducing the frequency and severity of “off” periods, and dyskinesias were significantly improved. The addition of stimulation to medication, which was significantly reduced only in the STN group, resulted in patients experiencing adequate mobility for a much larger proportion of the day along with a significant improvement in the ability to perform daily living activities [12].

DBS of STN or GPi showed a marked capacity to improve motor features in PD, further supporting the importance of the STN-GPi projection in the pathophysiology of PD. However, a comparison of the UPDRS-III scores in the “off” medication state obtained at baseline with the UPDRS-III scores of 3-4 years after DBS surgery in the “off” medication and off stimulation condition revealed no significant difference in either the STN or GPi treated groups. The only noticeable difference observed was that DBS of the STN conveys a significant reduction in L-DOPA daily dose that was not observed in patients in the GPi group. However, side effects were less frequently encountered in patients treated with stimulation of the GPi [12].

The PPN plays an important role in the initiation and maintenance of locomotion in experimental animals. Researchers have suggested that modulation of the activity of the PPN may be beneficial in treating gait disturbances and akinesia [13]. In a study of six patients with bilateral implantation of DBS electrodes in the STN and PPN in the “on” medication state, the association of STN-DBS and PPN-DBS provided a significant improvement when compared to the specific benefit mediated by the activation of either single target with PPN-DBS to be more effective on gait and postural problems [13]. The PPN is bounded on its lateral side by fibers of the medial lemniscus and its medial side by fibers of the superior cerebellar peduncle and its decussation [14]. The rostral pole of the PPN contacts the dorsomedial aspect of the posterolateral substantia nigra, while the most caudal pole of the PPN is adjacent to neurons of the locus coeruleus [14].

Glutamatergic inputs to the PPN from the STN have been described in rats, and inputs from the cervical and lumbar segments of the spinal cord to the area of the PPN have been shown in rats. These studies have suggested that cholinergic PPN neurons act as a relay station for the spinal cord sensory afferents to the thalamus; however, the neurotransmitter system involved in the spinal input is unknown. Three separate populations of neurons displaying rhythmic activity with locomotion can be recorded extracellularly in the area of the PPN in the decerebrate cat. Research studies have proposed that gait and posture abnormalities, in addition to rigidity and bradykinesia, may in part reflect the loss of neurons or the suppression of neuronal activity in the PPN. In PD, the inhibitory GABAergic projections from the GPi (and possibly the SNr) to the thalamus and PPN are overactive [14]. In a study involving three patients with advanced PD, PPN-DBS was described as improving REM sleep and cognition [15]. In addition, low-frequency PPN-DBS has been reported to improve L-DOPA-refractory freezing of gait (FOG) and reduce the frequency of falls in some patients. A 73-year-old male patient with a nine-year history of PD responded to L-DOPA for the first four years, then developed progressive medication refractory FOG. Bilateral PPN-DBS was provided six years after symptom onset with a good initial response with a significant reduction in FOG and falls. At the three-year postoperative clinic visit, the patient reported regaining a marked improvement in gait and balance by turning off the stimulation overnight and keeping it on only during the day [15]. In conclusion, the PPN remains an investigational target for DBS in patients with L-DOPA-refractory FOG [15]. The PPN may also have a role in SNc degeneration through an excitotoxic effect of glutamatergic synaptic contacts from the PPN onto dopaminergic SNc neurons [14].

Intraoperative neurophysiological signals monitored during installment of electrodes

PD is characterized by irregular electrophysiological activity, such as bursts and oscillatory activity. This can be used as a marker during DBS placement surgery in the STN. Using MER and MRI, a group of researchers was able to cross-validate the localization of permanently placed DBS electrodes. MER was used to identify the irregular firing of the STN; recordings lasting at least 45 seconds and showing spike clusters with a minimum peak-to-peak amplitude of 60 microvolts were included. The researchers then used MRI to verify the localization and trajectories used intraoperatively. The data collected found that spike amplitudes were higher in the limbic region of the STN than in the distal lateral STN [16].

Additionally, they found that the STN differed by subdivision by their beta oscillations; there was more beta activity in the sensorimotor and associative division than in the limbic division. The authors of this paper theorize that differences in beta oscillation and spike patterns could provide additional markers when attempting to localize electrode targets [16]. Stimulation of the STN (specifically the pars reticulata, which serves as an output to the basal ganglia) at low intensities, such as 14 Hz, did not modify the oscillatory activity of the STN neurons. However, high-frequency stimulation of 140 Hz produced a mean decrease in neuronal firing of 67%, demonstrating that high-frequency stimulation of the STN is more effective for treatment [17].

The GPi is another target for DBS electrode placement, and being a deep brain structure, it can present a serious surgical risk to the patient. The use of MER can help minimize risk and increase the accuracy of electrode placement. In a study aiming to analyze electrophysiological recordings of DBS GPi neuronal activity, Klempir found that 24 kHz signal processing, along with 0.5-5 kHz bandpass filtering and automated detection, resulted in being able to clearly distinguish the GPi from the structures surrounding it. Microelectrodes registered for single-unit recording were used to distinguish the neuronal activity of the GPi from its external segment and the other nearby structures [18].

DBS can provide patients with alleviation of Parkinsonian symptoms, epilepsy, and motor disorders. However, DBS is not without risks. As the name implies, DBS requires that the DBS electrode be placed deep within the brain, with the STN and globus pallidus being common electrode sites for permanent placement. The invasive nature of this surgery presents its risks, but in addition to those are risks of electrode burns and incorrect electrode placement. Improper electrode placement will not improve the quality of life for the patient but could also result in overstimulation or damage to other structures (such as motor spasticity due to internal capsule stimulation) [19]. Burns can typically be avoided by ensuring that the electrode has the proper impedance for the charge it will be conducting and by turning off the DBS system during high RF (radio frequency) energy exposure (such as MRI scanning). Transcranial electrical motor evoked potentials (TCeMEP) has long been used to monitor the functional integrity of nerves during spinal surgery, but since this type of IONM technique requires stimulating the cortex, there was not much literature regarding the use of TCeMEP on DBS patients. Srisooksai et al. found that TCeMEP could be safely carried out on DBS patients at the “lowest suitable stimulation voltage” [20]. The patient’s DBS system was also turned off during the procedure. Postoperatively, the patient was able to move on command, and the DBS system rebooted without issue or reset, demonstrating that TCeMEP can be safely carried out on DBS patients and that DBS patients may not need to be as limited in regards to procedures they can safely endure so long as precautions are taken first.

Advantages for intraoperative neurophysiological monitoring use in PD cases

Due to poor resolution from MRI scanning, MER (Microelectrode recording) was once a necessary part of DBS surgery to accurately place the electrode; the STN simply could not be visualized via MRI scan. This required moving the electrode down 1mm at a time until the burst firing of the STN is reached and stopped with the steady firing of the substantia nigra reticulata (SNr). However, with advancements in MRI technology, including scanners of 3+ Tesla, MER has become less of a “staple” during DBS surgery, and its efficacy has been called into question due to the increase in surgery time MER requires, as well as the risk of ICH (intracranial hemorrhage) [21]. A 2019 study looked at this variable (increased time) to test whether using MER helped or hindered STN DBS placement surgery. Twenty-four patients having undergone STN DBS surgery were reviewed. The average total time taken for the MER portion of the surgeries averaged 41.2 +/- 6.3 minutes, and though the number of electrodes used per side was not different, there was a difference in the procedure time per side. This study found that while STN DBS surgery with MER took longer than the same surgery without MER, patients still demonstrated outcomes consistent with the literature. The outcomes were not significantly better than had MER not been used in this study, but the authors cite another study in which the MER group demonstrated significantly better outcomes than the non-MER group, providing support that MER does have an impact on the clinical outcomes.

Additionally, though ICH is a risk associated with DBS surgery, this study had no cases in the cohort. Microelectrode also proves useful in adhering to selected trajectories, especially if CSF leakage has caused a brain shift, as in this paper. The shift decreased the placement percentage on the second side (75% vs 61%), respectively [21]. Though MER is not relied on as frequently due to advances in MRI technology, it provides real-time monitoring that can be used to prevent postoperative deficits. As well as this, the additional time needed to perform the recording does not increase the surgical risk undertaken to perform the procedure.

Microelectrode recording increases the accuracy of electrode placement. During DBS surgery, correct electrode placement is necessary to alleviate Parkinsonian symptoms, avoid overstimulation, and, in more severe cases, electrode burns over the deep brain structures. To mitigate these risks, microelectrode recording can detect how far the electrode is from the internal capsule. This can be accomplished by stimulating through the microelectrode, called micro-stimulation. The low voltage of the micro-stimulation can induce micro-stimulation-induced involuntary muscle contractions when close to the internal capsule, warning the surgical team [19]. Additionally, Mehanna et al. also found that capsular macro-stimulation (stimulation of the internal capsule) was decreased in cases in which capsular micro-stimulation was closely observed throughout the surgery [19].

Possible future treatment options for DBS

Focused ultrasound is an early-stage, non-invasive treatment that was recently FDA approved for the treatment of tremor-dominant PD as well as dyskinesia. This technique works by focusing beams of high-frequency energy on deep brain targets (Focused Ultrasound Foundation). This approach could be used for movement disorders to ablate tissue, such as the subthalamic nucleus. For treating Parkinson’s disease, focused ultrasound can temporarily disrupt the blood-brain barrier so that desired pharmacological agents may more readily cross the barrier. This technology is still in its infancy, and more research is needed to prove its efficacy. However, if shown to be effective, focused ultrasound could present patients with a low-cost and non-invasive treatment option over DBS [22].

Levodopa (L-DOPA), which is the precursor for dopamine, can help alleviate some of the symptoms of Parkinson’s, but unfortunately, this treatment loses its efficacy over long-term use, eventually rendering it ineffective as a treatment option. DBS has, especially in recent years, become the procedure of choice for treating PD over pharmacological treatment with L-DOPA. DBS does not have the dose-duration issue present with L-DOPA and has a demonstrated efficacy, as seen in the literature. However, DBS is highly invasive and not without risks (electrode burns, intracranial hemorrhage) [21]. Vibration has been demonstrated to correct pathological oscillatory activity by causing somatosensory evoked potentials (SSEPs) to fire in a pattern aligned with the oscillations of the stimuli [23]. A long-term study taking place over 12 weeks, using 21 participants, looked at the efficacy of 40 Hz vibration administered via a physio-acoustic therapy device, which allows for targeted vibration therapy. In comparing the control and experiment groups, only the experimental group demonstrated a significant improvement over their baseline, and all general motor symptoms improved. This response occurred in 16 of the 21 participants [23].

Many patients complain of the frustration they experience with their daily amount of “off” time hours where the medications are not working or when their symptoms are unresponsive to their current medication regimen. DBS typically improves “off” time by an average of 4-6 hours daily.

DBS may provide a form of treatment that makes their symptoms more manageable. DBS is an approach to interrupt and block the brain's irregular and uncoordinated impulse signaling, which can help lessen motor symptoms such as tremors, rigidity, stiffness, and bradykinesia. Unlike thalamotomy and pallidotomy, DBS utilizes a form of treatment that does not cause permanent damage to the brain tissue and is completely reversible. Thalamotomy and pallidotomy procedures surgically destroy tiny areas of the brain and are not reversible. However, DBS is reversible and does not cause permanent damage but rather provides a means to electrically stimulate certain areas of the basal ganglia to moderate motor symptoms associated with PD. Although DBS may significantly improve motor symptoms and a patient’s quality of life, it does not cure PD and cannot slow the progression of the disease. DBS is simply a designed form of treatment to help minimize “off” periods and maximize “on” periods in a patient’s daily lifestyle and routine.

DBS involves using electrodes implanted in subcortical areas that elicit electrical pulses directly to high populations of dopaminergic neurons. The most common target is the STN, but the GPi is also a target for DBS. Research studies have not found a significant difference in improvement between stimulation of the STN vs. stimulation of the GPi. However, clinical trials do suggest that DBS of the STN conveys a significant reduction in the daily dose of L-DOPA compared to DBS of the GPi. DBS of the STN has suggested that there may be sweet spots within the STN that provide hyper-direct cortical connectivity pathways to M1, SMA, and PFC. In additional research studies, the PPN may be a new target for DBS that helps treat locomotion problems associated with freezing gait and akinesia. Microelectrode (MER) recording and advanced MRI are used to ensure electrode placement accuracy. Intraoperatively, microelectrode recording is used to locate the target for placement. Using MER, stimulation of the STN at high frequencies (140<) decreased oscillatory neuronal firing by 67%. The current hypotheses on the mechanism of action associated with DBS include depolarization blockade, synaptic inhibition, synaptic depression, stimulation-induced disruption of pathological network activity, and stimulation of afferent axons projecting to the STN [24].

## Conclusions

DBS has been a beneficial treatment method for PD patients who are not responding well to their L-DOPA medications or have been experiencing significant “off” periods when their medications do not relieve their symptoms fast enough. Their symptoms worsen as patients experience reduced “on” time and increased “off” time. Patients with PD may take medications such as monoamine oxidase inhibitors, amantadine, dopamine agonists, and carbidopa-levodopa. The various medications can have variable effects on different patients concerning how fast these medications are absorbed into the body and start to provide relief against PD symptoms. DBS is currently used to treat severe motor fluctuations or tremors in advanced stages of PD with the STN or GPi as the primary nuclei to target in the stimulation. The data from previous clinical trials do not give one specific basal nuclei an advantage over the other in stimulation results; however, there may be additional nuclei to target in bilateral stimulation, such as the PPN.
